# Pyorrhœa Alveolaris Due Largely to Systemic Predisposition

**Published:** 1892-04

**Authors:** C. N. Peirce


### International Dental Journal.

Vol. XIII. April, 1892. No. 4.

### Original Communications.[1]

### PYORRHŒA ALVEOLARIS DUE LARGELY TO SYSTEMIC PREDISPOSITION.[2]

BY C. N. PEIRCE, D.D.S.

A fact well recognized is that heredity and adaptation are important factors in the production and modification of organs as well as of organisms, and that when these are once established in harmony with a comparatively stable environment, they become so fixed that they are, through successive generations, reproduced with comparatively limited variations. So when deviations from the recognized forms occur, we may naturally look for the factor which has been instrumental in causing this divergence; and in doing so endeavor to place it, when found, either in the line of ancestral traits, in one of those modifications arising from effort of adaptation to a new environment, or to a new use or a disuse of the parts, or to the influence of some specific poison with which the patient or individual has been recently inoculated.

Though man, in his ancestral line, far exceeds the limits of definite knowledge, he is evidently still much younger or more recent

[1] The editor and publishers are not responsible for the views of authors of papers published in this department, nor for any claim to novelty, or otherwise, that may be made by them. No papers will be received for this department that have appeared in any other journal published in the country.

[2] Read at a meeting of the Odontological Society of Pennsylvania, February 13, 1892.

in origin than many other mammals, but yet old enough to have become so fixed in his prominent physical characteristics that each individual of succeeding generations is hopefully expected to be not unlike the ancestral type. But, notwithstanding this hereditary and adaptive influence, there are constantly appearing local minor modifications, peculiarities, or abnormalities, which, wherever located, are such an expression as to interfere with either symmetry, function, comfort, or that economy for which nature is so constantly struggling.

The etiology of such abnormal manifestations is a matter of deep and continued interest and anxiety to the physiologist, as well as the pathologist, for to successfully anticipate, combat, or correct these unfavorable conditions, this knowledge of their etiology is of the greatest importance.

While, as above expressed, they are in position local, the inquiry comes with much force, are they wholly due to traumatic influences, or are they the local expression of some systemic condition, and located in the special field or district, because of a predisposition of the tissues, structures, or organs involved? There are several such abnormalities to which reference might be made, but in order not to confuse the subject for this evening's consideration, one only shall receive attention at the present time, and that one in its progress and results is so familiar to you all that I need simply name it. The tissues involved are the alveolus, the alveolo-pericemental membrane, and the cementum of the root. And while the results of pyorrhœa alveolaris are so disastrous, yet it is usually regarded as a purely local affection, both in origin and influence. There are, however, some associate lesions usually accompanying it, so that it may, with a good degree of appropriateness, be estimated as being largely under the direction of a systemic predisposition. In recording the history of its progress I shall endeavor to note the evidence in favor of this theory. The structure of the dense tissues involved is too well known and appreciated to need repetition; but a few words regarding the soft or more vascular structures may not be out of place.

This tissue, with its blood-vessels and nerves, which connects the cementum of the tooth and the inner wall of the alveolus with the arterial and nervous systems we shall designate, as above, the alveolo-pericemental membrane; its office is regarded as functional, physical, and sensory, and it is so arranged as to closely invest the root of the tooth and fill the space between it and its alveolus; protecting the cementum of the root at times beyond the alveolar border up to its union with the enamel, and below the apical end to the limit of its bony surrounding. It is composed of fibres which are firmly fixed to the cementum and from that surface pass out in a slightly divergent direction, lying nearly parallel with each other, save the deviations which occur to give place to the blood-vessels, nerves, and lymphatics, to be embedded in the alveolus, and thus help to hold the tooth in position; osteoblasts which build up portions of the alveolar walls; cementoblasts which construct the cementum; fibroblasts for the augmentation or renewal of its fibrous tissues, and osteoclasts and cementoclasts for the removal of these tissues when stimulated by either physiological or pathological considerations,—all have a physiological importance. This membrane varies in thickness in different individuals, and in the same individual at different periods of life, or under different systemic or local conditions. The abnormalities to which it is liable are such as are common to all vascular tissues, but are much exalted in severity and results in consequence of the membrane being located between two unyielding surfaces. This root-membrane Charles S. Tomes describes as a "connective tissue of moderate density, devoid of elastic fibres, and richly supplied with nerves and blood-vessels." The fact that the "principal fibres" run across from cementum to process without break in continuity demonstrates it to be but a single membrane, so that one for the root and another for the alveolus is not a feasible proposition. On its inner aspect, where it becomes interwoven with the cementum, it is pictured by histologists as consisting of a fine net-work of interlacing bands, which are firmly adherent to the cementum. This surface, it is alleged by Tomes and Black, is richly cellular, containing large, soft, nucleated plasm-masses, which are the cementoblasts for the formation of cementum, and which, by their offshoots or prolongations, communicate with vascular masses within the cementum. The vascular supply of this membrane is derived from three sources,—the vessels in the gums or soft tissue covering the alveolus, the vessels in the bone, and branches of the vessels destined for the pulp-supply, the last being by far the most important and especially worthy of note, as will be seen from a subsequent paragraph. So please keep in mind that the tooth-pulp and the tissue, which is now our root-membrane, receive their blood-supply largely from the same source. Blood enters the tooth-pulp in great abundance; the dentine through the tubules is also well supplied, and these tubules, by prolongations, connect with cement-corpuscles or lacunæ, and these again are, through a multiplicity of little canals and vessels, brought into close connection or intimate relation with the vascular membrane on the root. So between the pulp inside and the cemental membrane on the outside, there is in a normal tooth a continual interchange of living plasma, in addition to the supply which it receives from the vessels of the gum and alveolus. With this brief description of the alveolo-pericemental membrane, the tissue, the abnormal diseased condition of which is under consideration, we proceed to the origin and progress of its deviations from health. Dr. G. V. Black has very appropriately given us two terms to express the pathological conditions which are now known as pyorrhœa alveolaris, or "Riggs' disease," gingivitis, or *phagedenic pericementitis* and calcic inflammation, or better, *calcic pericementitis*. Pyorrhœa alveolaris simply expresses a flow of pus from the alveolus, and could as well be applied to acute alveolar abscess as to these chronic conditions. Of the two conditions designated by Dr. Black, it is our purpose to speak of one only,—*calcic pericementitis*. Gingivitis or phagedenic pericementitis may or may not be a systemic expression, though it would be quite safe to so regard it; while the other, calcic pericementitis, is regarded by many as a purely local affection, and yielding, if at all, to local treatment only. To those holding such views let one inquiry be made. Are renal and hepatic incrustations or accumulations to be regarded as wholly the result of abnormal conditions of the liver and kidney, or are they not rather due to some systemic condition wherein nutrition, appropriation, absorption, and waste are involved? Is it not a process of transformation, which is characterized by the deposit in these tissues or organs of various salts derived directly from the blood? We all know that calcareous deposition takes place in special localities or organs as a normal physiological process, as in the formation of bones and teeth; but when found in excess, or from these fields it is diverted and elsewhere deposited as an amorphous salt instead of an organized structure, and with a truly pathological result, shall it be stated with any degree of consistency or wisdom that the localities receiving it are the only unfaithful or treacherous members? I trust not. There may be foreign substances, living or dead, necrosed particles, trichinæ, shot, etc., which are sources of irritation, and which, for the protection of surrounding tissues, may become encysted. We may also have excementosis persistently progressing to the extent of greatly enlarging the transverse diameter of the root, but in such cases there is a local stimuli sufficient to excite the cell and call into play its power of multiplication without seriously disturbing its function of reproduction; but these instances are widely different from renal and hepatic depositions, as well as from the serumal deposits upon the teeth, all of which, though only precipitates, at times become so extremely hard they cannot be cut with a knife, and yet they are only an amorphous or unorganized salt. Hepatic depositions rarely occur before middle life; out of four hundred cases collected by Hein, less than four per cent. were under twenty-five. The same could with truth be said of serumal deposits on the teeth, the larger proportion of cases showing no manifestation until after the age of forty years.

Morbid changes in the secretions, sedentary habits of life, errors in diet, are noted by Fried. Theod. Frerichs as being favorable to the formation of gall-stones, while a calculous diathesis is spoken of in connection with both urinary and biliary calculi. Ziegler, in his work on pathological anatomy, says, "concretions of calcium phosphate and carbonate deposited in the kidney constitute what is called calcareous infiltration." "It occurs chiefly in aged persons in whom resorption of the bony structures is active; but it may occur without such resorption."

While we recognize that biliary and urinary deposits are not identical in analysis with serumal or sanguinary deposits upon the roots of teeth, yet the urinary have in common with the serumal or sanguinary deposit a marked concomitant, "a calculous diathesis," and not infrequently where the former urinary deposit is recognized the latter is also found.

In over seventy-five per cent. of the cases of calcic pericementitis under my care,—and I have had, and now have, a large number, —induration of tooth tissue has preceded serumal deposit.

This *antecedent* is to me an important feature in the diagnosis and prognosis of the case. This filling up of tubuli and reduction of pulp-chamber and canals establishes the diathesis, and at the same time necessarily reduces very materially the blood-supply to the pulp, and, in consequence thereof, places the pericemental membrane for the time in a hyperæmic condition. We have already admitted the intimate blood-relation existing between these two vascular tissues. This change of tooth-structure we recognize as the first physical deviation from normality, with subsequent ones depending upon predispositions due to inherited or acquired tendencies.

This second deviation from normality we now recognize as occurring in the alveolo-pericemental membrane, and we designate it as a hyperæmic condition, showing itself visually and mechanically. To the eye there is more or less reddening and turgescence on the gum, irritation of continuity, while the tooth itself is, in many cases, perceptibly elongated to give room for the congested and necessarily thickened membrane from the dilatation of the blood-vessels of the part and their repletion with blood.

This hyperæmic state may be described as being either active or passive; but in the conditions under consideration we shall, from results, assume that it was first active, inasmuch as we have an increased flow of blood to the parts inducing congestion, and passive to the extent of a reduced or diminished flow of blood from the part producing engorgment, over-distention of the vessels, to be relieved only by exudation; this increased quantity of liquid plasma transudes under pressure from the vessels. Let it be here recognized that this transuding plasma is that portion of the blood which carries the mineral element, the accumulation of which on the cementum of the root is at once patent in response to both its source and to its unavoidable presence. I repeat, unavoidable, for this transudation from the blood, with its calcic freight, is far in excess of the capacity of the lymphatics to carry away. So, from stasis of the blood we have stasis of lymph, with a saturation of the surrounding tissues far in excess of the normal degree. Shall we term this an œdematous condition, œdema of engorgment? Collateral œdema it is, for it is the effect of some previous condition.

Inflammation is now localized and present in full force,—redness, swelling, heat, and some pain, with a marked impairment, and in some cases the total arrest, of the function of the tissue involved. The conditions established are vascular disturbance, disordered nutrition, an accumulation of exuded plasma, an arrest of function, and a precipitation of calcic crystals in the shape of an amorphous salt on the surface of the root, which, while remaining in this position, reacts so as to exalt and continue the inflammatory condition. Now, what shall we consider the primary *noxious* element which causes an alteration in the vessels, disturbs or arrests their normal function, and excites inflammation? Is it possible that this injurious agent is brought to this locality in the blood itself? Can it be that this calcic material, which was first appropriated in solidifying the tooth, is now increased in such quantity as to be the cause of these clogged and altered vessels and of other conditions which are doing such irreparable damage, or is it only a sequence? To ascertain a correct explanation of these abnormalities these inquiries are made. One concomitant of it all must certainly be admitted, the presence of a "calculous diathesis."

Another, and not the least important, condition is yet to be noted. The exudation has reached or assumed a purulent form,—

a whitish, milky, or yellow appearance,—and is now called "matter," or pus. This change has doubtless been through the influence of bacteria, infectious micro-organisms. The tendency of these organisms is to increase the breaking down or dissolution of the tissues, so that we now have formed a pus-containing cavity—an *abscess*—a *pocket*—from which, if without treatment, pus is continually discharged until the tissues are destroyed and the tooth drops out, or, to relieve discomfort, is artificially removed. In some instances, when left unattended, exudation undergoes putrefaction, and is offensive. The staphylococcus (so called from being in close cluster, like grapes) thrives, says Carl Frankel, at ordinary room-temperature, but multiplies far more luxuriantly when at a higher point. It *is not* a harmless concomitant of purulent inflammatory processes, as has been demonstrated by successful transmissions; these experiments proving without doubt its septic character.

We have given, as far as possible, a history of the origin, progress, and termination of calcic pericementitis, or pyorrhœa alveolaris, so termed.

The reasons for estimating it as being associated with a systemic predisposition are,—

*First.* It has invariably, as an antecedent, a calculous diathesis.

*Second.* The age at which it becomes manifest is in correspondence with similar renal and hepatic affections, rarely being under forty years.

*Third.* When treated to a comparatively successful termination, it needs constant watchfulness and care, else a few months will see it as active and virulent as in any previous stage.

*Fourth.* While under proper and efficient care with marked satisfactory progress, systemic disturbances with a limited period of neglect will not only retard repair, but will greatly exalt the inflammatory condition with all its accompaniments.

*Fifth.* Never is one tooth alone the victim of the disease, but organs on both sides of the mouth, as well as in both the inferior and superior jaws, extending ofttimes to the whole denture, are probable victims; and when once located on any tooth, not one in the mouth is free from the possibility of attack at a near or remote period.

*Sixth.* The great frequency at which it can be traced directly to an ancestral influence; fully ninety per cent. are of this character, and I deem it one of the strongest evidences in favor of its being the result of a constitutional condition.

*Seventh.* Ordinary salivary calculus may be present to a greater or less degree, even to the complete visual obliteration of all the teeth in the mouth; but when this is thoroughly removed, the trifling irritation at the gingival borders yields at once to the application of appropriate remedies, providing the serumal or sanguinary deposit is not present.

The treatment to be pursued is too familiar to the profession to need repetition.

